# TRIM28 mediates Mettl5 ubiquitination to promotes Th2 polarization

**DOI:** 10.3389/fimmu.2025.1524633

**Published:** 2025-05-05

**Authors:** Beiping Miao, Lihua Mo, Shihan Miao, Xiwen Zhang, Haoyue Zheng, Yixuan Dong, Bailing Xie, Yuanyi Zhang, Yun Liao, Yu Liu, Ping Tang, Pingchang Yang

**Affiliations:** ^1^ Department of Otolaryngology, Head & Neck Surgery, Shenzhen Second People’s Hospital and Shenzhen Clinical Medical Research Center for Otolaryngology Diseases, Shenzhen, China; ^2^ Department of General Practice Medicine, Third Affiliated Hospital of Shenzhen University and Luohu Clinical College of Shantou University Medical College, Shenzhen, China; ^3^ State Key Laboratory of Respiratory Diseases Allergy Division at Shenzhen University and Institute of Allergy & Immunology, Shenzhen University School of Medicine, Shenzhen Key Laboratory of Allergy & Immunology, Shenzhen, China; ^4^ Department of Immunology & Key Laboratory of Tropical Translational Medicine of Ministry of Education & Department of Immunology, School of Basic Medicine and Life Sciences, Hainan Medical University, Haikou, China

**Keywords:** allergy, th2, immunity, inflammation, GATA3

## Abstract

**Background:**

Th2 polarization is the primary characteristic of airway allergy (AA) and many other immune disorders. Further elucidation of its mechanism is necessary. The immune cells of patients with immune diseases have been found to have abnormal epigenetic status. This research intends to examine the role of methyltransferase-like 5 (Mettl5) in regulating homeostasis in CD4^+^ T cells.

**Methods:**

An AA mouse model was established with dust mite extracts as a specific antigen. The epigenetic marks in the *Gata3* gene of CD4^+^ T cells were evaluated using chromatin immunoprecipitation assay and cross-enzyme-linked immunosorbent assay.

**Results:**

Spontaneous airway Th2 polarization was observed in mice carrying Mettl5-deficient CD4^+^ T cells. The quantity of Mettl5 was decreased in airway CD4^+^ T cells of AA mice, which was negatively correlated with the AA response. Hyperubiquitination was detected in Mettl5 in airway CD4^+^ T cells of AA mice, which was negatively correlated with hypomethylation status at the *Gata3* promoter and the high transcription activity of the *Gata3* gene. The elevated quantity of TRIM28 was detected in airway CD4^+^ T cells of AA mice. The presence of TRIM28 induced Mettle protein ubiquitination and degradation in CD4^+^ T cells. Inhibition of TRIM28 reconciled the Mettl5 activity and *Gata3* gene transcription in airway CD4^+^ T cells of AA mice, and attenuated AA.

**Conclusions:**

Low Mettl5 levels in airway CD4^+^ T cells resulted in Th2 polarization. Inhibition of TRIM28 restored the levels of Mettl5 in airway CD4^+^ T cells, and suppressed experimental AA.

## Introduction

Th2 polarization indicates that more than needed numbers of Th2 cells gather in the local tissues. The local tissues are saturated with the excess amounts of Th2 cytokines produced by Th2 cells. As a consequence, Th2 pattern inflammation is induced in the local tissues (1). The overproduction of Th2 cells can induce the production of IgE by plasma cells. IgE makes mast cells/basophils sensitized. Re-exposure to specific antigens induces mast cells to release allergic mediators, which triggers allergy attacks (2). Th2 polarization is also implicated in the progression of various immune disorders, including autoimmune diseases (3). The development of Th2 polarization is still not fully understood. Remedies used to alleviate Th2 polarization-related diseases are currently limited (1). Thus, it is urgent to in depth investigate the mechanism by which Th2 polarization develops. New remedies that can be used to treat Th2 polarization-related disorders are necessary.

It is known that the over production of Th2 cytokines is the essential factor in Th2 pattern inflammation such as airway allergy (AA) (1). The overexpression of Th2 cytokines can be a key factor in the development of Th2 polarization (4). In general, the gene transcription is controlled by the methylation machinery. Hypermethylation suppresses gene transcription. Hypomethylation promotes gene transcription (5). There have been reports that indicate both hypermethylation and hypomethylation are involved in the pathogenesis of AA, including allergic rhinitis and asthma (6). Both methylases and demethylases are involved in regulating the methylation status of the genes in immune cells of subjects with AA.

Methyltransferase-like 5 (Mettl5) is a methylase, which plays an essential role in RNA modification, specifically N6-methylation (m^6^A) (7). Published data show that Mettl5 mediated m6A mainly focuses on cancer progression (8). Reports also mention Mettl5 is involved in the pathogenesis of autoimmune disorders (9) and nervous development (10). It is reported that Mettl5 promotes immune cell infiltration (11, 12), which is a major pathological feature of AA (13, 14). Whether Mettl5 is involved in the pathogenesis of Th2 polarization and AA is to be further investigated. Tripartite motif-containing protein 28 (TRIM28) plays a role in the development of effector CD4^+^ T cells (15). TRIM28 is a ubiquitin E3 ligase. It can initiate or promote the ubiquitination and degradation of targeted proteins. In this study, we found a decrease in the amount of Metll5 at the Gata3 promoter and a high amount of TRIM28 in airway CD4^+^ T cells of mice with AA. Mettl5 was crucial in maintaining the homeostasis of the *Gata3* gene. TRIM28 induced Mettl5 ubiquitination and degradation to promote the *Gata3* transcription, which contributed to Th2 polarization.

## Results

### Spontaneous Th2 polarization is detected in the airways of mice carrying the *Mettl5* gene deficient CD4^+^ T cells

**Figure 1:**
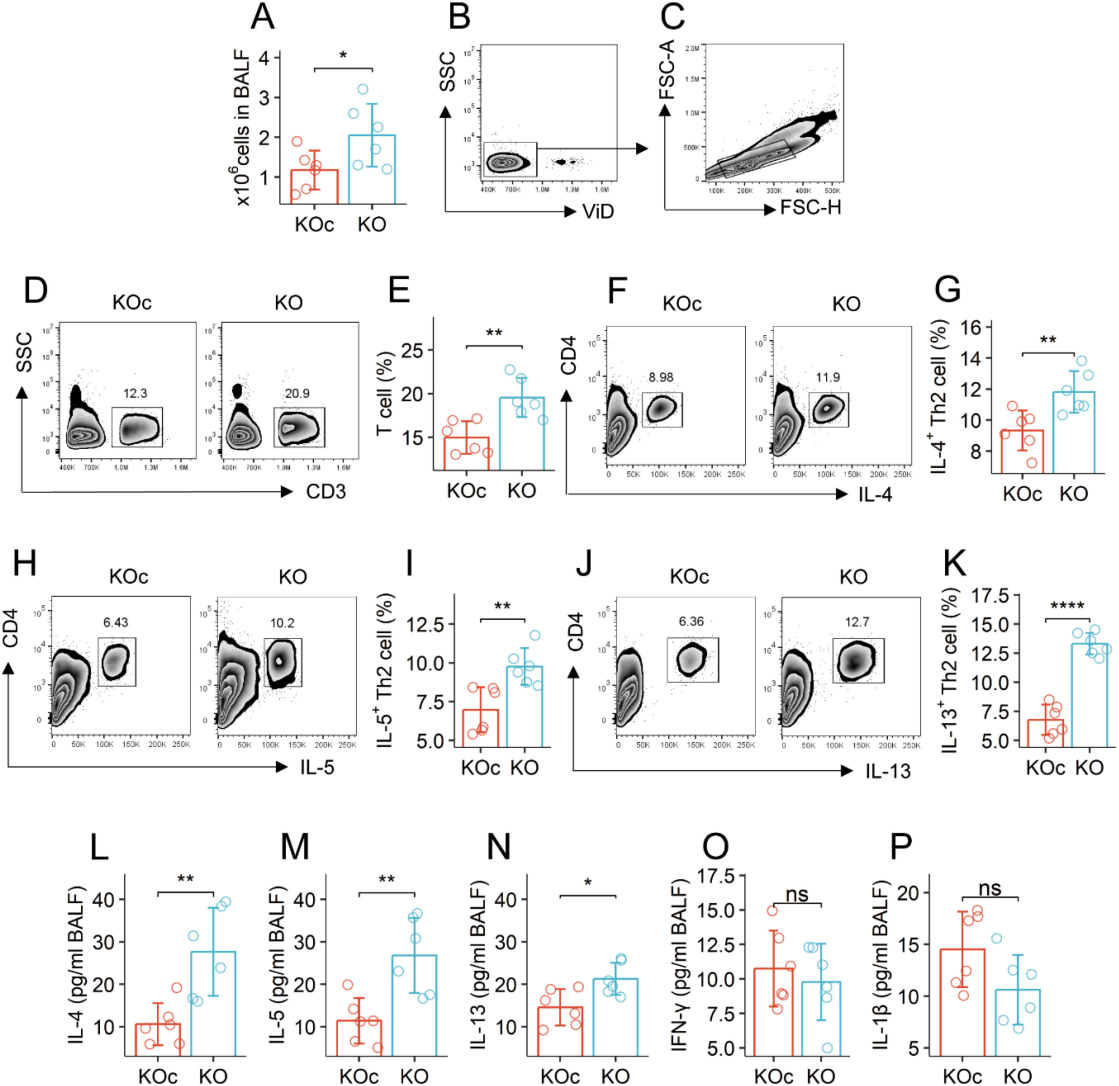
KO mice have spontaneous airway Th2 polarization. BALF was collected from KOc mice and KO mice, and analyzed by flow cytometry and ELISA. **(A)** total cell counts of BALF. **(B, C)**, gating strategy. **(D-K)** gated plots show indicated cell types on the Y axis. Bars show counts of indicated cells. **(L–P)** bars show the quantities of indicated cytokines on the Y axis. The data of bars are presented as mean ± SD. Each bubble in bars presents one sample (tested in triplicate). Statistics: Student’s *t*-test. *p<0.05; **p<0.01; ****p<0.0001; ns, Not significant. Each group consists of 6 mice. Each experiment was repeated 3 times. KOc: *Mettl5* ^f/f^ mice. KO: *Mettl5* ^f/f^ *Cd4* ^CreERT2^ mice.

The total cells, frequencies of T cells, and Th2 cells were significantly more in the bronchoalveolar lavage fluid (BALF) collected from the mouse strain carrying the *Mettl5* gene deficient CD4^+^ T cells (*Mettl5* ^f/f^ *Cd4* ^CreERT2^ mice) than those from *Mettl5* ^f/f^ mice (the control mice) (**Figures 1A–K**). The quantities of Th2 cytokines (IL-4, IL-5, and IL-13) in BALF collected from *Mettl5* ^f/f^ *Cd4* ^CreERT2^ mice were higher than those in *Mettl5* ^f/f^ mice (**Figures 1L–N**). The levels of Th1 cytokines in BALF were slightly lower in the *Mettl5* ^f/f^ *Cd4* ^CreERT2^ mice than in the *Mettl5* ^f/f^ mice, but not enough to reach the significant criterion (**Figures 1O, P**). The results indicate that the *Mettl5* ^f/f^ *Cd4* ^CreERT2^ mice have spontaneous Th2 polarization in the airways.

### Sensitization-suppressed Mettl5 in CD4^+^ T cells links to airway Th2 polarization

**Figure 2:**
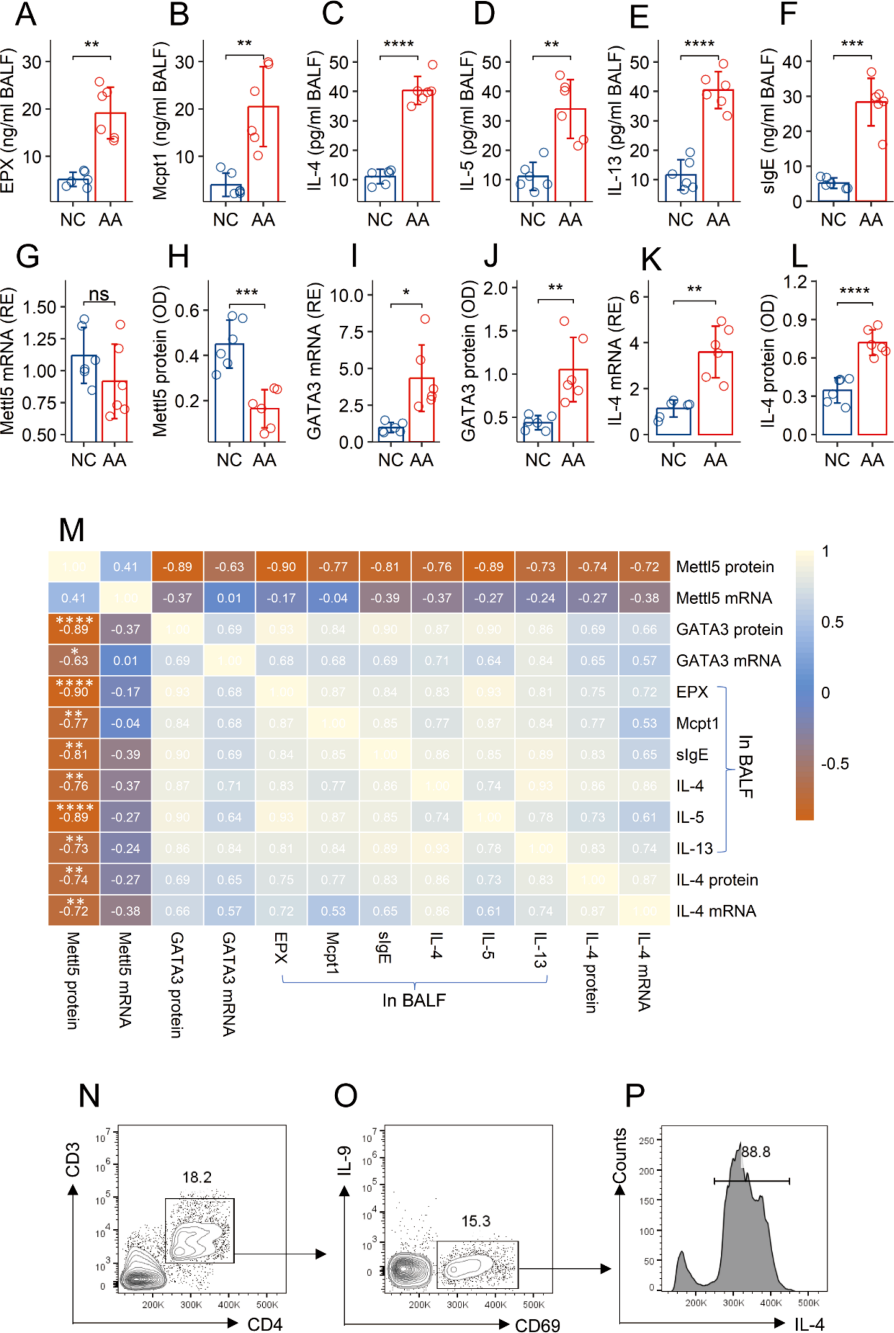
Assessment of the correlation between Mettl5 and AA response. An AA mouse model was established. **(A–F)** bars show the quantities of indicated molecules (denoted on the Y axis) in BALF. **(G–L)** CD69^+^CD4^+^ T cells were isolated from the lung tissues. Bars show the quantities of indicated molecules in cellular extracts. M, a heatmap shows the coefficients between the indicated items. **(N–P)** CD3^+^CD4^+^ T cells were gated from single cells isolated from the lung tissues **(N)**, from which CD69^+^ cells were gated **(O)**; **(P)** the histogram shows IL-4^+^ cells from CD69^+^ T cells. The data of bars are presented as mean ± SD. Each bubble in bars presents one sample (tested in triplicate). Statistics: Student’s *t*-test **(A–L)** and Pearson correlation coefficient test **(M)**. *p<0.05; **p<0.01; ***p<0.001; ****p<0.0001. Each group consists of 6 mice. Each experiment was repeated 3 times.

A mouse AA model was established. The mice showed an AA response, manifesting elevated quantities of allergic mediators, Th2 cytokines, and specific IgE (sIgE) in BALF (**Figures 2A–F**). It is reported that CD69^+^CD4^+^ T cells are involved in allergic attacks (16, 17). CD69 is a marker of cell activation. Thus, CD69^+^CD4^+^ T cells could be the antigen specific CD4^+^ T cells in subjects after a challenge with specific antigens (18). CD69^+^CD4^+^ T cells were isolated from the lung tissues, and analyzed by RT-qPCR and ELISA. A decrease in Mettl5 protein (but not its mRNA), an increase in GATA3 and IL-4 were found in CD69^+^CD4^+^ T cells, which were significantly different between AA mice and naïve control (NC) mice (**Figures 2G–L**). A correlation assay was conducted with the data. The quantity of Mettl5 protein in CD69^+^CD4^+^ T cells was negatively correlated with the parameters of the AA response. The GATA3 levels in CD69^+^CD4^+^ T cells were positively correlated with the AA response. A negative correlation was detected between the data of Mettl5 protein and GATA3 (**Figure 2M**). The data indicate that the levels of Mettl5 were lower in airway CD69^+^CD4^+^ T cells, which may be associated with the regulation of GATA3 in CD4^+^ T cells, and the pathogenesis of AA. Additionally, the expression of IL-9 was below the detectable levels in airway CD69^+^CD4^+^ T cells (**Figures 2N–P**).

### Abnormal methylation status at the *Gata3* promoter of airway CD4^+^ T cells in AA mice

**Figure 3:**
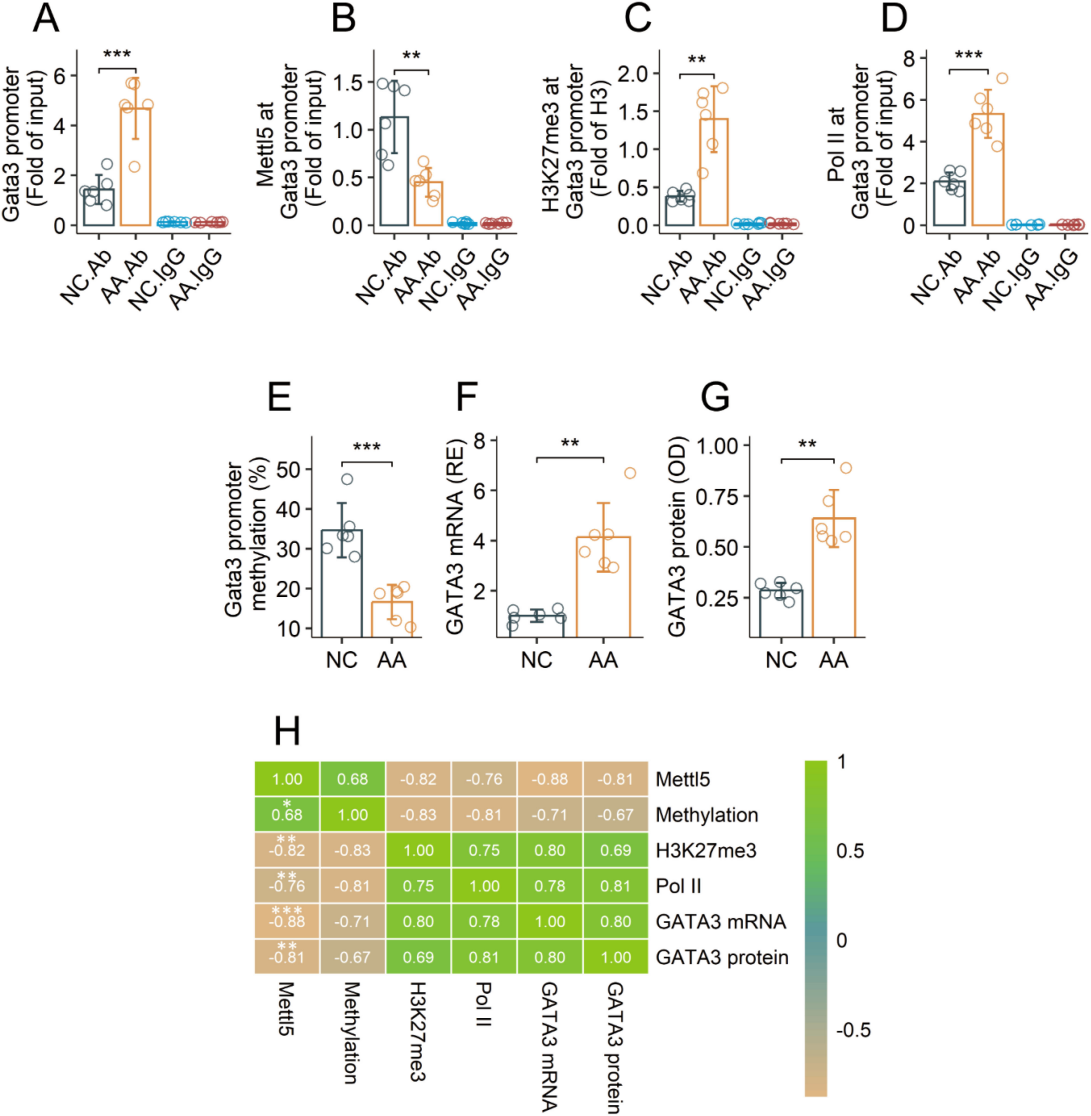
Assessment of the activities of the *Gata3* promoter in airway CD69^+^CD4^+^ T cells in AA mice. Airway CD69^+^CD4^+^ T cells were isolated from AA mice and NC mice, and analyzed by ChIP assay. **(A)** quantities of *Gata3* promoter DNA in ChIP products. **(B, C)** quantities of Mettl5 and H3K27me3 at the *Gata3* promoter. **(D)** the *Gata3* promoter methylation status. **(E)** quantity of Pol II at the *Gata3* promoter. **(F, G)** expression of GATA3. **(H)** correlation coefficients between indicated items. The data of bars are presented as mean ± SD. Each bubble in bars presents one sample (tested in triplicate). Statistics: Student’s *t*-test **(A–G)** and Pearson correlation coefficient test **(H)**. **p<0.01; ***p<0.001. IgG, Isotype IgG (used as a negative control Ab in ChIP assay). Ab, Abs used in ChIP assay. Each group consists of 6 mice. Each experiment was repeated 3 times.

Airway CD69^+^CD4^+^ T cells were isolated from AA mice and NC mice, and analyzed with chromatin immunoprecipitation assay (ChIP). Using a HuR Ab as a bait (HuR can bind to the *Gata3* gene (19, 20), the *Gata3* promoter was identified in ChIP products (**Figure 3A**). The same ChIP products showed the presence of Mettl5 and H3K27me3. The quantity of Mettl5 was lower and the quantity of H3K27me3 was higher in samples from AA mice as compared to samples from NC mice (**Figures 3B, C**). Hypomethylation was also found in the *Gata3* promoter DNA, and a significant difference was detected between samples from NC mice and the AA mice (**Figure 3D**). The gene transcription activities (indicated by the levels of RNA polymerase II, Pol II) at the Gata3 promoter were much higher in the AA samples than that in the NC samples (**Figure 3E**). The quantities of GATA3 in the AA group were also significantly higher than those in the NC group (**Figures 3F, G**). A correlation test revealed a strong correlation between Mettl5 and Gata3 promoter methylation. Negative correlation was detected between Mettl5 and H3K27me3 or Pol II at the *Gata3* promoter, or the expression of GATA3 in airway CD69^+^CD4^+^ T cells isolated from AA mice (**Figure 3H**). The results indicate that the methylation status at the Gata3 promoter in airway CD69^+^CD4^+^ T cells of AA mice is abnormal.

### Hyperubiquitination causes low levels of Mettl5 in CD4^+^ T cells of AA mice

**Figure 4:**
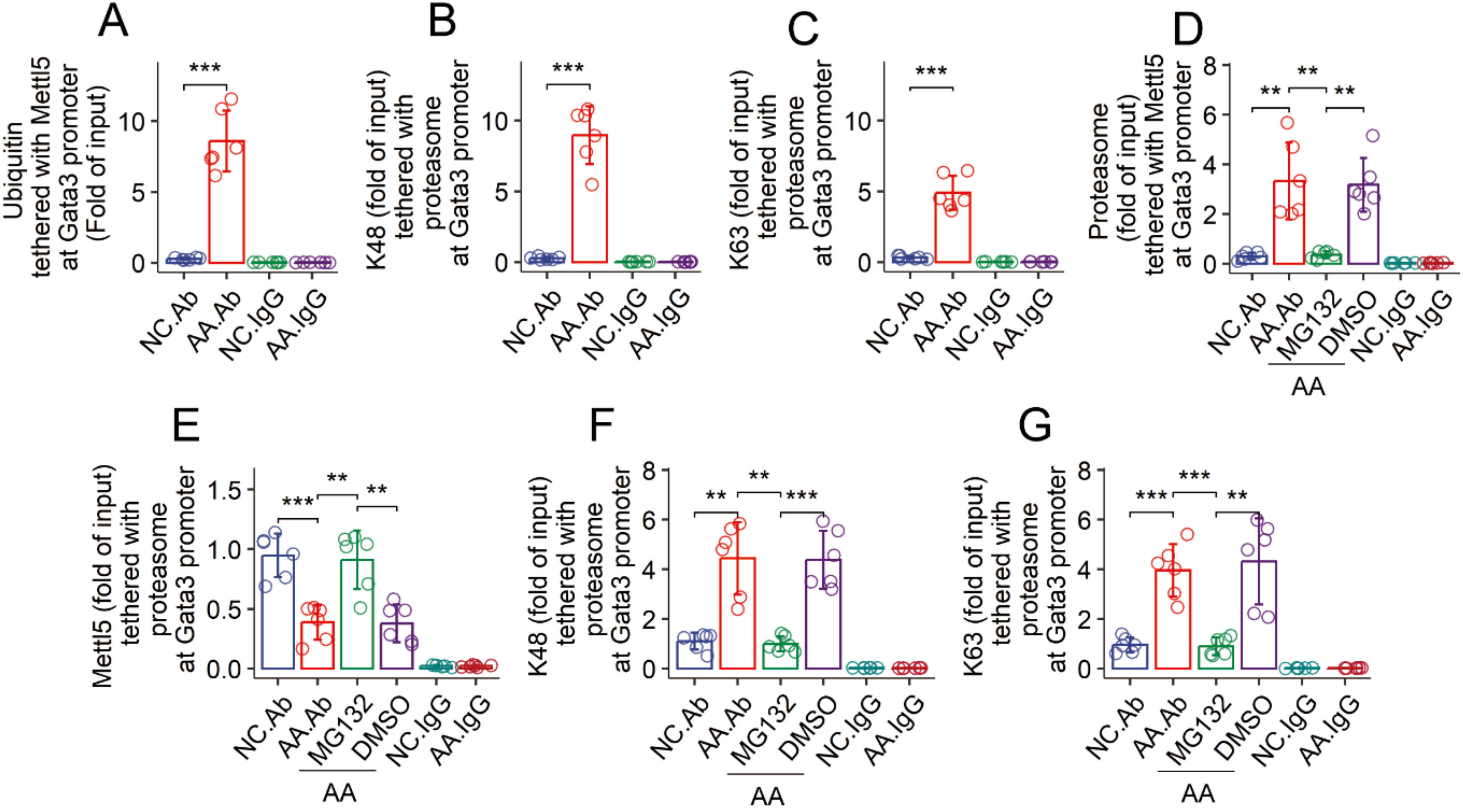
Assessment of the ubiquitination status of Mettl5 in CD4^+^ T cells. **(A–C)** airway CD69^+^CD4^+^ T cells were isolated from NC mice and AA mice. The cells were analyzed by ChIP-cross-ELISA. Bars show the quantities of indicated molecules together with Mettl5 at the *Gata3* promoter. **(C–G)** the treatment for mice is denoted on the X axis. MG132 (DMSO): Mice were treated with nasal instillations (containing MG132, 20 μM, or DMSO) daily in the period of sensitization. Bars show the proteasome complexes at the *Gata3* promoter of airway CD69^+^CD4^+^ T cells. The data of bars are presented as mean ± SD. Each bubble in bars presents one sample (tested in triplicate). Statistics: ANOVA + Tukey HSD test. **p<0.01; ***p<0.001. Each group consists of 6 mice. Each experiment was repeated 3 times. IgG, Isotype IgG (used as a negative control Ab in ChIP assay).

Data reported above indicate that Mettl5 was modified post transcriptionally. It implicates that Mettl5 in airway CD4^+^ T cells of AA mice may be degraded abnormally. Ubiquitination is a primary mechanism for protein degradation. Thus, airway CD69^+^CD4^+^ T cells were prepared from AA mice and NC mice, and analyzed by ChIP that targeting to the *Gata3* promoter using an HuR Ab as a guide (19, 20). The ChIP products were analyzed by cross-ELISA. We found that samples from AA mice showed elevated quantity of ubiquitin (**Figure 4A**), K48 and K63 in Mettl5 protein at the *Gata3* promoter (**Figures 4B, C**). The results suggest that Mettl5 at the *Gata3* promoter is at hyperubiquitination status in airway CD69^+^CD4^+^ T cells of AA mice. Mettl5 was detected in the proteasomes of airway CD69^+^CD4^+^ T cells of AA mice, which was reduced by nasal administration of MG132 during the sensitization period (**Figures 4D, E**). Meanwhile, we also found that the administration of MG132 reduced the quantities of K48 and K63 in the proteasome of airway CD69^+^CD4^+^ T cells in AA mice (**Figures 4F, G**). The findings indicate that Mettl5 in the *Gata3* promoter is in a hyperubiquitination state.

### TRIM28 mediates Mettl5 ubiquitination

**Figure 5:**
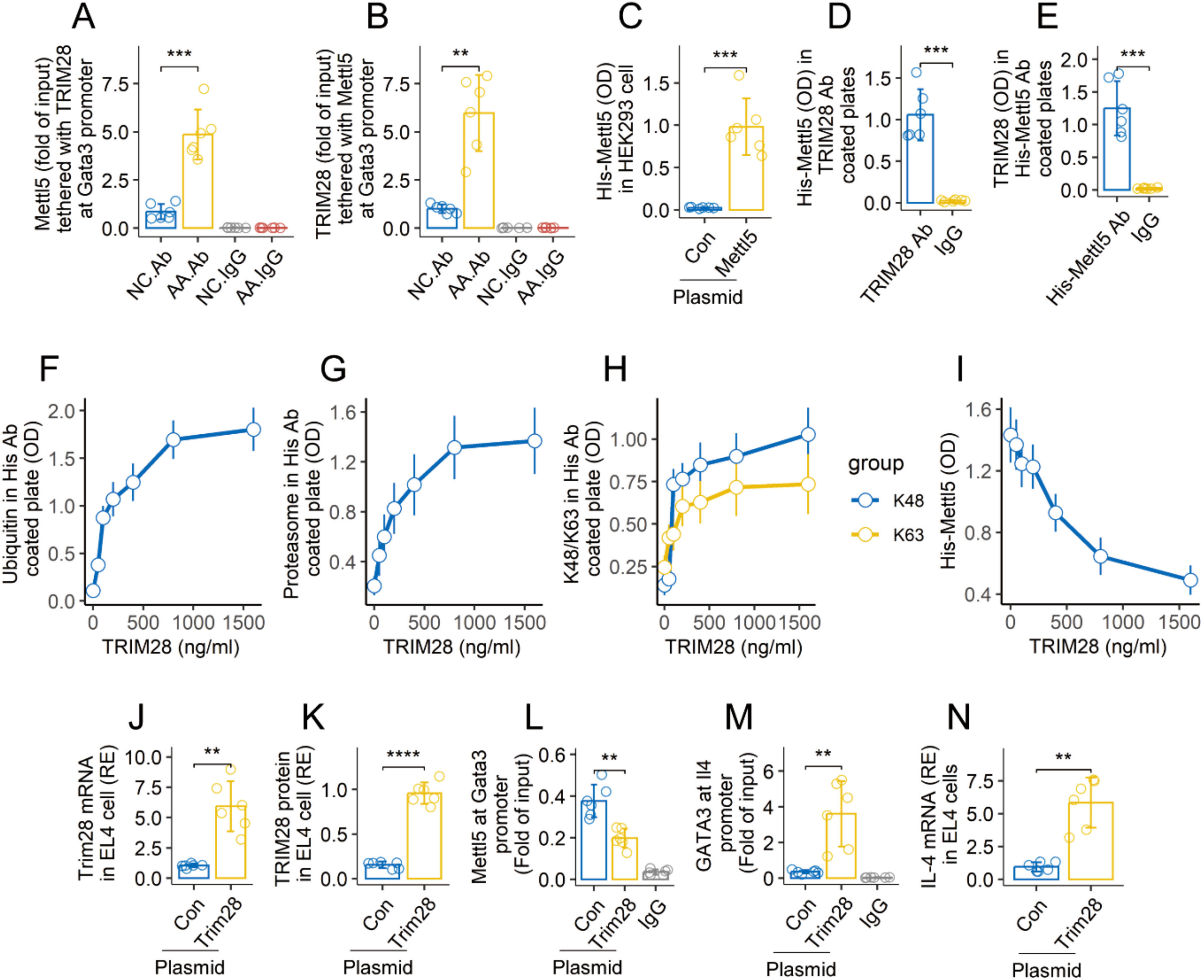
TRIM28 induces Mettl5 ubiquitination and degradation. **(A, B)**, airway CD69^+^CD4^+^ T cells were isolated from AA mice and NC mice. The cells were analyzed using ChIP-cross-ELISA. Bars show a complex of Mettl5 and TRIM28. **(C)** recombinant Mettl5 protein in HEK293 cells. **(D, E)** Mettl5-expression HEK293 cells were exposed to TRIM28 (1 μg/ml) in culture overnight. Bars show a complex of TRIM28 and His-Mettl5. **(F–I)** Mettl5-expression HEK293 cells were exposed to TRIM28 at gradient concentrations overnight. Cellular extracts of HEK293 cells were analyzed using cross-ELISA. The line plots show the quantities of indicated items. **(J, K)**, enforced expression of TRIM28 in EL4 cells. **(L)** quantity of Mettl5 at the *Gata3* promoter in EL4 cells. **(M)** quantity of GATA3 at the *Il4* promoter in EL4 cells. **(N)** expression of IL-4 in EL4 cells. The data of bars are presented as mean ± SD. Each bubble in bars presents one sample (tested in triplicate). Statistics: ANOVA + Tukey HSD test **(A, B)** and Student’s *t*-test **(C–E)**. **p<0.01; ***p<0.001; ****p<0.0001. The experiments of line plots were repeated 3 times. Ab in **(A, B)**: Anti-HuR Ab (used in ChIP assay to anchor the *Gata3* promoter). IgG, Isotype IgG (used as a negative control Ab in ChIP assay). Con, Control plasmid. Each group consists of 6 mice. Each experiment was repeated 3 times.

Published data indicate that TRIM28 is involved in the development of effector CD4^+^ T cells (Gehrmann, 2019 #4). We wondered if TRIM28 is also involved in Mettl5 ubiquitination and degradation in airway CD69^+^CD4^+^ T cells of AA mice. The *Gata3*-targeted ChIP products were analyzed by cross-ELISA with Abs of TRIM28 and Mettl5. The *Gata3* promoter was found to possess a complex of TRIM28 and Mettl5 as shown in the results (**Figures 5A, B**). TRIM28 is a ubiquitin E3 ligase. The data imply that the physical contact between TRIM28 and Mettl5 may cause Mettl5 ubiquitination and degradation. To test this, HEK293 cells were transfected with plasmids expressing His-Mettl5. The recombinant Mettl5 proteins were detected in HEK293 cells 48 h after the transfection (**Figure 5C**). The cells were then exposed to exogenous TRIM28 in culture overnight. Cellular extracts were prepared from the HEK293 cells, and analyzed using cross-ELISA. A complex of TRIM28 and His-Mettl5 was detected (**Figures 5D, E**). Then, the HEK293 cells that produce His-Mettl5 were exposed to TRIM28 in culture at gradient doses overnight. This resulted in enhanced ubiquitination of His-Mettl5, elevated proteasome, K48 and K63 in His-Mettl5 in a TRIM28 dose-dependent manner (**Figures 5F–H**). The quantity of His-Mettl5 in HEK293 cells was also reduced (**Figure 5I**). Furthermore, we prepared the enforced expression of TRIM28 in EL4 cells (**Figures 5J, K**). It resulted in a decrease in Mettl5 at the Gata3 promoter, and an increase in GATA3 at the Il4 promoter in EL4 cells (**Figures 5L, M**). Elevated expression of IL-4 was found in the EL4 cells (**Figure 5N**). The findings confirmed that TRIM28 triggers Mettl5 ubiquitination and degradation, resulting in an increase in IL-4 expression.

### Inhibition of TRIM28 rescues Mettl5 ubiquitination, reconciles airway Th2 polarization, and mitigates AA

**Figure 6:**
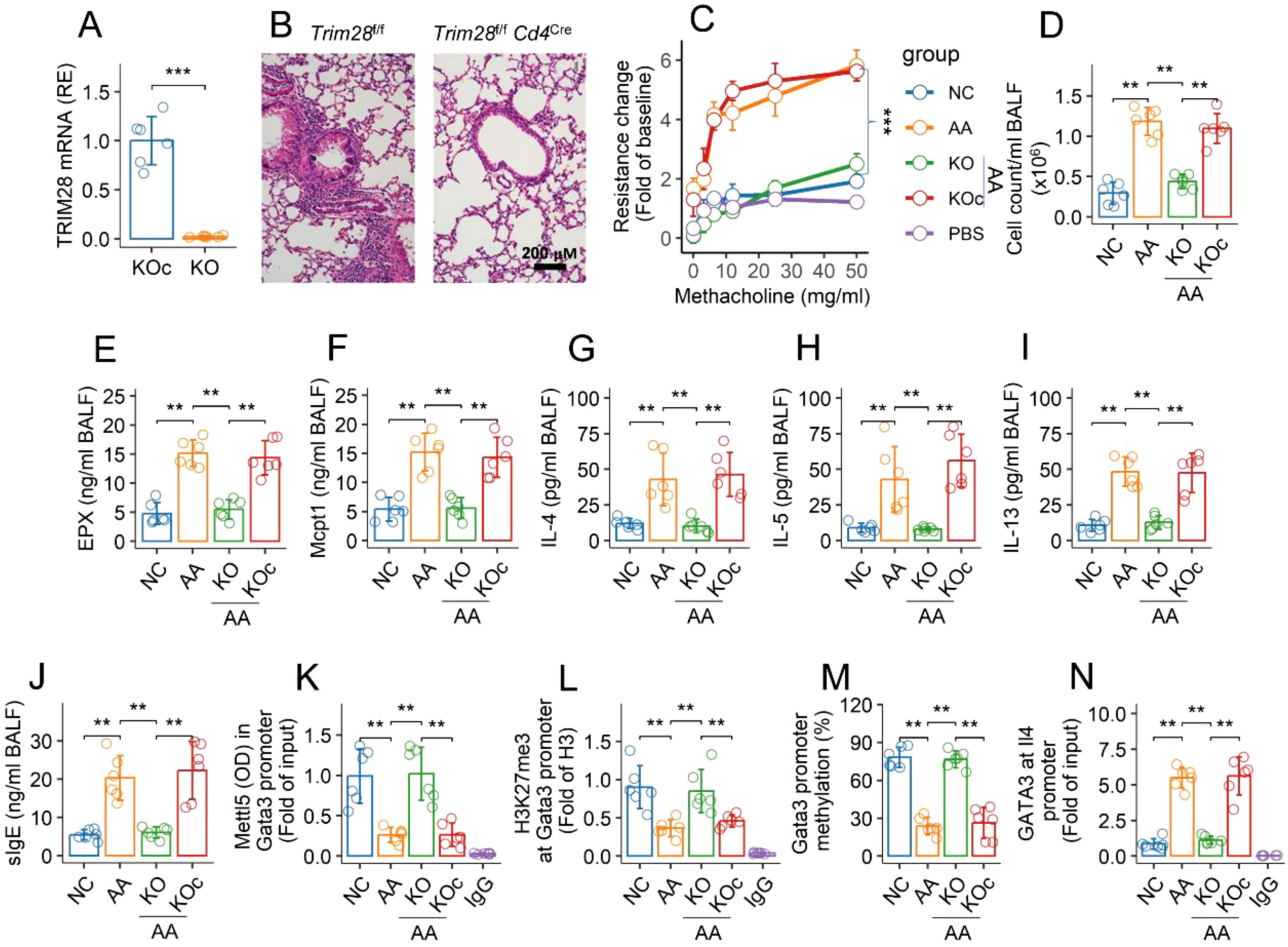
Inhibition of TRIM28 reconciles epigenetic abnormality at Gata3 gene of airway CD4^+^ T cells and mitigates AA. **(A)** quantity of TRIM28 mRNA in KOc mice and KO mice. **(B)** representative histology images of the lungs (×200). **(C)** mouse airway resistance changes in response to challenge with methacholine. **(D)** total cell counts in BALF. **(E–J)** quantities of indicated molecules in BALF. **(K, L)** airway CD69^+^CD4^+^ T cells were isolated from NC mice and AA mice. The cells were analyzed using ChIP-cross-ELISA. Bars show the quantities of indicated items at the Gata3 promoter. **(M)** bars show percentage of Gata3 promoter methylation. **(N)** bars show quantity of GATA3 at the *Il4* promoter. The data of bars are presented as mean ± SD. Each bubble in bars presents one sample (tested in triplicate). Statistics: Student’s *t*-test **(A)** and ANOVA + Tukey test **(C–N)**. **p<0.01; ***p<0.001. KO mice, *Trim28* ^f/f^ *Cd4* ^Cre^ mice; KOc mice, *Trim28* ^f/f^ mice; IgG, Isotype IgG (used as a negative control Ab in ChIP assay). Each group consists of 6 mice. Each experiment was repeated 3 times.

An AA mouse model was established with a mouse strain carrying conditional *Trim28*-deficient CD4^+^ T cells (*Trim28* ^f/f^ *Cd4* ^CreERT2^ mice) (**Figure 6A**). The mice showed an AA response, including lung inflammation (**Figure 1B**), airway hyperresponsiveness (**Figure 6C**), infiltration of inflammatory cells (**Figure 6D**), elevated quantities of allergic mediators (EPX and Mcpt1) (**Figures 6E, F**), Th2 cytokines (**Figures 6G–I**), and specific IgE (sIgE) (**Figure 6J**) in bronchoalveolar lavage fluid (BALF). Tamoxifen activation of the CreERT2-loxP system led to an increase in Mettl5 in the *Gata3* promoter region, reconciliation of airway Th2 polarization, and mitigation of the AA response (**Figures 6B–J**). We also found that inhibition of Trim28 resulted in an increase in the quantity of Mettl5 and H3K27me3 at the Gata3 promoter (**Figures 6K, L**). The methylation of the *Gata3* promoter was up regulated (**Figure 6M**). The quantity of GATA3 was reduced at the *Il4* promoter (**Figure 6N**). The results demonstrate that regulation of TRIM28 can mitigate experimental AA and reconcile the epigenetic abnormality of the *Gata3* gene in airway CD69^+^CD4^+^ T cells of AA mice.

## Discussion

This study revealed that the TRIM28-Mettl5 signaling pathway had a significant impact on the regulation of GATA3 expression in CD4^+^ T cells. TRIM28 induced Mettl5 ubiquitination at the *Gata3* promoter, which resulted in the *Gata3* gene demethylation and transcription. GATA3 is the critical transcription factor of IL-4. As a consequence, the expression of IL-4 was increased in CD4^+^ T cells. The data indicate that the TRIM28-Mettl5 signaling pathway plays a critical role in the airway Th2 polarization.

The data show a decrease in Mettl5 at the *Gata3* promoter of antigen specific CD4^+^ T cells in the airways of AA mice. The quantity of Mettl5 in CD4^+^ T cells has a negative correlation with the AA response. The data indicate a link between Mettl5 and the pathogenesis of AA. The decrease in Mettl5 levels at the Gata3 promoter in CD4^+^ T cells may contribute to the development of AA. The inference was verified by using *Mettl5* ^f/f^ *Cd4* ^CreERT2^ mice in this study. The airways of this mouse strain showed spontaneous Th2 polarization. Others also noted that Mettl5 was involved in the development of Th2 cells (11). Mettl5 has the ability to promote inflammatory cell infiltration (12), which is a significant characteristic of AA. Our findings indicate that BALF has profound inflammation cell infiltration, which is significantly greater in AA mice than in NC mice. Wang and his colleagues concluded that Mettl5 has a strong link to the immune regulation of immune modulators, chemokines, and their receptors in the immune microenvironment (21). Current data demonstrate that Mett5 can be localized at the *Gata3* promoter. It is much less in AA mice compared to NC mice. The above information suggests that it is necessary to find its significance in the pathogenesis of AA.

Mettl5 is an enzyme that catalyzes methylation. Its essential function is to induce or facilitate methylation in targeted items. Previous studies found that Mettl5 is a methyltransferase of N^6^-methyladenosine (m^6^A) (22). It has been reported that cardiac hypertrophy can be accelerated by loss of Mettl5 through epitranscriptomic regulation of SUZ12 expression (23). The Myc pathway is inhibited to downregulate PD-L1 expression and immune escape of hepatocellular carcinoma cells when Mettl5 is knocked down, as found by Xu and colleagues (24). Our data show another aspect of Mettl5. Current data show that Mettl5 at the *Gata3* promoter of CD4^+^ T cells is necessary to maintain immune homeostasis in the airways because ablation of Mettl5 results in spontaneous Th2 polarization in the airways. The polarization of Th2 cells is a critical pathological feature of AA. This suggests that loss or insufficient Mettl5 in CD4^+^ T cells may be one of the factors that facilitate the development of AA.

Current data show that Mettl5 is localized at the *Gata3* promoter. A positive correlation was detected between Mettl5 and the *Gata3* promoter methylation. It suggests that Mettl5 plays a role in the induction methylation or maintenance of the methylation status at the *Gata3* promoter. On the other hand, it indicates that the loss or insufficient Mettl5 may result in the demethylation of the Gata3 promoter. Our data support this inference by showing decreased methylation status of the *Gata3* promoter methylation as well as a decrease in H3K27me3 at the *Gata3* promoter. In general, the demethylation of a gene is accompanied with its transcription (25). Our data also supports this notion. We discovered a high level of gene transcription activity at the *Gata3* gene. Taken together, the data suggest that Mettl5 maintains the methylation status of the Gata3 promoter in CD4^+^ T cells. This proposal is supported by further experimental data. Ablation of the Mettl5 gene in CD4^+^ T cells resulted in spontaneous Th2 polarization in the airways as we observed in the present study.

A decrease in the quantity of Mettl5 was observed in CD4^+^ T cells of AA mice. Ubiquitination and degradation are the primary mechanisms for protein degradation (26). The data show that ubiquitin was colocalized with Mettl5 at the *Gata3* promoter of airway CD4^+^ T cells of AA mice. It suggests that Mettl5 experienced ubiquitination. The fact implicates the degradation of Mettl5. It was verified by the findings of elevated proteasome, K48 and K63 in the same location. It results in an increase in GATA3 expression in CD4^+^ T cells. The elevation of GATA3 is a common event in allergic diseases including AA. For example, Palikhe and colleagues report that the expression of GATA3 in CD4^+^ T cells is much higher in severe asthma patients than in those with mild asthma (27). It is also significantly higher in allergic asthma patients than in normal control subjects (28). The present data show that the expression of GATA3 can be regulated by epigenetic activities. Mettl5 fulfills the duty of maintaining the methylation status of the Gata3 gene in CD4 T cells. However, it can be regulated. Hyperubiquitination can reduce the amount of Mettl5, which results in an increase in the *Gata3* expression.

Based on the above information, it is significant to identify the causal factors for the decrease in Mettl5 in CD4^+^ T cells. Our data show an increase in the expression of TRIM28 in CD4^+^ T cells of AA mice. Previous reports indicate that TRIM28 is involved in the development of effector CD4^+^ T cells (15). TRIM28 is an E3 ligase. It induces ubiquitination in targeted molecules (29). The current data suggests that TRIM28 is responsible for Mettl5’s ubiquitination and degradation. As a consequence, the expression of GATA3 is increased. The results suggest that the inhibition of TRIM28 may be an option to reduce the expression of GATA3. Previous reports indicate that cell-surface β2 adrenergic receptor activation can suppress the expression of GATA3 in CD4^+^ T cells (30). Whether activation of β2 adrenergic receptor can suppress the expression of TRIM28 and indirectly reduce the expression of GATA3 is to be investigated. Others found directly induced GATA3 ubiquitination by Sox (SRY-related high-mobility-group (HMG)-box)-12 also suppressed the expression of GATA3 in Th2 cells (31). On the other hand, as TRIM28 has broad epigenetic regulatory effects, the off-target possibility needs to be considered in the plan to use it as a therapeutic agent for related diseases.

The data show that the conditional ablation of TRIM28 can efficiently suppress experimental AA. The results suggest that to develop inhibitors of TRIM28 has the translation potential to be used for the treatment of AA and other allergic diseases. T cells are not absorbing cells. Exogenous drugs are not easy to penetrate into T cells to regulate the expression of TRIM28. This is a challenge that requires further investigation.

It is known that m^6^A 1832 18S rRNA modification is specifically mediated by Mettl5. It is crucial for mRNA maturation, stability, and function exertion. Dysregulation of m^6^A is linked to various diseases (32). Deregulation of m^6^A is associated with the pathogenesis of AA (33). Current data show that Mettl5 can be also found in the *Gata3* promoter region. Whether m^6^A modification by Mettl5 is also involved in the GATA3 dysfunction-related diseases, such as allergic disorders, is an interested topic, and needs to be further investigated.

Additionally, published data indicate that the invariant natural killer cells can be promoted by IL-4; those cells can also produce IL-4 (34, 35). Our data indicate that Mettl5 is involved in the regulation of IL-4. Whether Mettl5 is also involved in regulation of invariant natural killer cells is an interesting topic to be further investigated. Also, the data emphasize CD69^+^ CD4^+^ T cells in the lung, but it remains unclear whether Mettl5–TRIM28 interactions are cell-type specific or also occur in other T helper subsets or innate lymphoid cells in the lungs or other organs; this needs to be elucidated in further studies. On the other hand, there are more questions to be answered: could METTL5 be recruited indirectly through protein–protein interactions, rather than forming a bona fide DNA–protein interaction? Does METTL5 contain any domains capable of directly interacting with histones or transcription factor complexes, or might it bind nascent RNA transcripts near the promoter, thereby appearing as though it binds DNA? These questions need to be further studied in the future.

In summary, the present data show that Mettl5 plays an important role in maintaining the epigenetic homeostasis of the *Gata3* gene in CD4^+^ T cells. Sensitization-induced TRIM28 can induce Mettl5 ubiquitination and degradation, which results in overexpression of GATA3, which contributes to airway Th2 polarization.

## Materials and methods

### Reagents

TRIM28 protein, enzyme-linked immunosorbent assay (ELISA) kits of eosinophil peroxidase (EPX), mast cell protease-1 (Mcpt1), IL-4, IL-5, IL-13, interferon (IFN)-γ, IL-1β, TRIM28, ubiquitin and GATA3 were purchased from Dakewe BioMart (Shenzhen, China). Antibodies (Abs) of H3K27me3 (ab6002), K48 (EP8589), K63 (EP8590-448), ubiquitin (EPR8590) was purchased from abcam (Waltham, MA). Abs of CD3 (sc-20047, AF488), CD4 (sc-19641, AF546), CD69 (sc-373799, AF594), CD19 (sc-390244, AF700), F4/80 (sc-377009, AF488), EMBP (sc-365701, AF594), HuR (sc-5261), GATA3 (sc-514427), TRIM28 (sc-136102), H3 (sc-517576), Pol II (sc-47701), proteasome (sc-166073) were purchased from Santa Cruz Biotech (Santa Cruz, CA). Mettl5 Ab (orb1255645) was purchased from biorbyt (Durham, NC). The purchase of materials and reagents for chromatin immunoprecipitation and RT-qPCR was made by Invitrogen (Carlsbad, CA).

### Mice

Male BALB/c mice were provided by Guangdong Experimental Animal Center in Fushan, China. *Mettl5* ^f/f^ mice, *Trim28* ^f/f^ mice, and *Cd4* ^CreERT2^ mice were purchased from Jackson Laboratory (Bar Harbor, ME). *Mettl5* ^f/f^ *Cd4* ^CreERT2^ mice were generated in-house by crossing *Mettl5* ^f/f^ mice and *Cd4* ^CreERT2^ mice. *Trim28* ^f/f^ *Cd4* ^CreERT2^ mice were generated by crossing *Trim28* ^f/f^ mice, and *Cd4* ^CreERT2^ mice. After five generations, mice were used in experiments. Mice were fed tamoxifen (3 mg/mouse in 0.3 ml corn oil (36)) daily for five consecutive days prior to experiments to activate the Cre-loxP system. Mice were housed in a facility that was free of pathogens. Food and water were freely accessible for all mice. Our university’s Animal Ethics Committee approved the use of mice in this study (approval number: A202300061). ARRIVAL’s guidelines were adhered to during the animal experiments.

### Airway single cell preparation

The lungs were excised from mice upon the sacrifice, cut into small pieces, and incubated with collagenase IV (0.5 mg/ml) and DNase I (0.2 mg/ml) for 30 min at 37°C with mild agitation. Single cells were filtered through a cell strainer, and cultured for further experiments.

### Cell culture

RPMI1640 medium was used to culture cells. Fetal bovine serum (10%), streptomycin (0.1 mg/ml), penicillin (100 U/ml), and L-glutamine (2 mM) were added to the medium. Cell viability was assessed using a Trypan blue exclusion assay. It was between 98.5-99.5%.

### Collection of bronchoalveolar lavage fluid

Upon sacrifice, 1 mL of saline was introduced into the lung of the mice using a syringe. The saline was promptly retrieved. It was repeated two more times. The lavage fluids collected at the three times were pooled, and used in further experiments. Total cell number was counted using a hemacytometer.

### Flow cytometry

The procedures of flow cytometry in a previous report (37) were followed in the present study. Briefly, single cells (10^6^ cells/sample) were stained with fluorescence-labeled Abs (Ab types are detailed in figures) or isotype IgG for 30 min at 4°C through surface staining or/and intracellular staining. The cells were analyzed using a flow cytometer (BD FACSCanto II). The data were processed using a software package (Flowjo) with the data obtained from isotype IgG staining as a gating reference.

### Isolation of CD4^+^CD69^+^ T cells

Single cells were prepared from the lung tissues of AA mice and naïve control (NC) mice as described above. Cells were stained with fluorescence-labelled Abs of CD3, CD4, and CD69, and sorted using a BD Aria flow cytometer. CD3^+^ cells were gated first, after which CD4^+^ and CD69^+^ T cells were gated and sorted. Cell purity was checked using flow cytometry. If the purity was not at or above 90%, isolation procedures were repeated.

### Enzyme-linked immunosorbent assay

Proteins in BALF, cell culture supernatant, and cellular extracts were evaluated by ELISA using commercial reagent kits following the protocols provided by the manufacturers.

### Real-time quantitative RT-PCR

RNA samples were extracted from cells collected from experiments, and converted to cDNA using a reverse transcription kit following the protocols provided by manufacturer. The cDNA samples were amplified in a Bio Rad CFX96 qPCR device using a reagent kit of SYBR green mast mix. The primers used in the present study include: Mettl5 (attgaaaacaaagcggttgc and gtcccaaagggaggattcat), GATA3 (ctggaggaggaacgctaatg and cagggatgacatgtgtctgg), TRIM28 (agtaccagctcaggcttgga and tccaggtggaagcaaaattc), and IL-4 (tcaacccccagctagttgtc and tgttcttcgttgctgtgagg). The results were processed using the formula of 2-ΔΔCt and presented as relative expression (RE) against the housekeeping gene Actb (agccatgtacgtagccatcc and ctctcagctgtggtggtgaa).

### Chromatin immunoprecipitation

Cells were collected from relevant experiments, fixed with 1% formalin for 15 min, and lysed with a radioimmunoprecipitation (RIPA) buffer. The lysates were sonicated to break the DNA into small pieces. The pre-existing immunocomplexes in the lysates were pre-cleared by incubating them with protein G beads for 2 hours. After centrifugation, the beads were discarded. A portion of the samples was collected and used as the input. Samples were incubated with Abs of HuR [to anchor the *Gata3* promoter (19, 20)] or GATA3 (to anchor the *Il4* promoter) at a concentration of 0.5 μg/ml overnight. The formed immunocomplexes were adsorbed by incubating with protein G beads for 2 h. The DNA-proteins on the beads were eluted using an eluting buffer. The DNA part was analyzed using qPCR in the presence of a pair of primers of the *Gata3* promoter (agccagggctacacagagaa and aaggaagtggcaaagcaaga) or *Il4* promoter (aggccagccagagctacata and cacagaagccagaggtgtca). The protein part was analyzed by cross-ELISA to assess the quantities of proteins of interest (detailed in figures). The results were presented as a fold of the input.

### Cross-ELISA

Cross-ELISA is used to identify the components of a protein complex. The procedure for cross-ELISA was similar to regular ELISA, except that the microplates were coated with a different Ab of interest. For example, to determine the amount of ubiquitin in Mettl5, the plate was coated with an Ab of Mettl5 (1 μg/ml) overnight. After blocking with 1% bovine serum albumin (BSA) for 30 min, a sample from the ChIP product or input (diluted to 1 µg/ml) was added to the plate, and incubated overnight. After washing, an Ab of ubiquitin was added to the plate. The rest procedures are the same as regular ELISA.

### Assessment of Mettl5 ubiquitination at the *Gata3* promoter

The protein portion of the ChIP assay from CD69^+^CD4^+^ T cells and HEK293 cells were prepared. The samples were analyzed by cross-ELISA to determine Mettl5 ubiquitination as described above.

### Assessment of *Gata3* promoter methylation

Following reported procedures (38), DNA samples were extracted from purified airway CD69^+^CD4^+^ T cells collected from experiments using a Universal Genomic DNA Extraction Kit provided by Invitrogen in Carlsbad, CA. Following the manufacturer’s protocol, the samples were processed using a bisulfide reagent kit (EpiJET; ThermoFisher). The samples were analyzed using qPCR. The primers of the *Gata3* promoter used in the experiments included a pair of methylation primers (aggaaaaattttagttttgttgttt and aactaccctacctctctacctctac) and a pair of unmethylated primers (tagttttgttgtttcggaagtttac and taccctacctctctacctctacgat). The results were presented as a percentage of Gata3 promoter methylation.

### Enforcing *Trim28* or *Mettl5* expression in HEK293 cells or EL4 cells

The *Trim28* gene was cloned into pcDNA3.1 vector to construct pcDNA3.1‐*Trim28* by Sangong Biotech (Shanghai, China). pcDNA3.1‐*Trim28* plasmids were transferred into EL4 cells using a Lipofectamine2000 reagent kit (Yita Biotech, Beijing, China) with the aid of electroporation. These procedures were also used in the enforcing *Mettl5* expression in EL4 cells. The pcDNA3.1-*Mettl5* plasmid was also provided by Sangong Biotech.

### Establishment of an AA mouse model

Randomly grouped BALB/c mice, *Trim28* ^f/f^ *Cd4* ^Cre^ mice, and *Trim28* ^f/f^ mice (6 mice/group) were sensitized to dust mite extracts (DME) following published procedures (39). Briefly, mice received a subcutaneous injection of DME (0.1 mg/mouse mixed in 0.1 ml alum) on day 1 and day 7, respectively. The induced immune response was boosted by treating mice with nasal instillations (20 μl/nostril, containing 5 mg DME/ml) daily from day 9 to day 22. Mice were challenged with a large dose of DME (20 μl/nostril, containing 50 DME/ml) on day 23.

### AA response assessment

After the challenge, the mice were sacrificed by cervical dislocation. BALF was collection as described above. A piece of the lung tissue was fixed with 4% formalin to process paraffin sections. Hematoxylin and eosin were used to stain the sections, and they were observed under a light microscope. Total cell numbers in BALF were counted using a hemacytometer. Quantities of EPX, Mcpt1, IL-4, IL-5, IL-13, and specific IgE in BALF were determined using ELISA.

### Statistics

The difference between two groups was determined by a Student’s *t*-test. ANOVA followed by a Tukey HSD test was conducted for multiple comparisons. Correlation between groups was determined using the Pearson correlation coefficient test. *p*<0.05 was set as a significant criterion.

## Data Availability

The original contributions presented in the study are included in the article/supplementary material. Further inquiries can be directed to the corresponding author/s.
